# Monitoring pain during use of virtual reality in debridement procedures of vascular wounds in outpatient care settings

**DOI:** 10.3389/fmed.2025.1658190

**Published:** 2025-12-03

**Authors:** Joanna Przybek-Mita, Dariusz Bazaliński, Anna Surmacz, Anna Kołodziej, Julia Bryła

**Affiliations:** 1 Institute of Nursing, Faculty of Health Sciences and Psychology, Collegium Medicum, University of Rzeszów, Rzeszów, Poland; 2 Laboratory for Innovative Research in Nursing, University Center for Research and Development in Health Sciences, Faculty of Health Sciences and Psychology, Collegium Medicum, University of Rzeszów, Rzeszów, Poland; 3 Specialist Hospital, Podkarpackie Oncology Center of Fr. B. Markiewicz in Brzozów, Brzozów, Poland; 4 Clinical Hospital, Fryderyk Chopin University, Rzeszów, Poland; 5 Student Scientific Circle of Elderly Care, Institute of Nursing, Faculty of Health Sciences and Psychology, Collegium Medicum, University of Rzeszów, Rzeszów, Poland

**Keywords:** wound debridement, virtual reality, pain monitoring, nurse, wound cleaning, outpatient care

## Abstract

**Introduction:**

Virtual Reality (VR) is an advanced technological system which, besides its use in entertainment and education, has been permanently introduced over the past quarter-century into physical and psychiatric rehabilitation as well as medicine. Its effectiveness has been demonstrated in pain management during medical and rehabilitation procedures in burn patients, alleviation of cancer pain, and reduction of labor pain. VR is increasingly used during routine medical procedures in children, such as blood sampling, intravenous cannulation, and vaccination, and holds promise for chronic pain management.

**Objective:**

The aim of this study was to assess pain during the treatment of hard-to-heal wounds of vascular origin using VR as an adjunct non-pharmacological pain therapy.

**Materials and methods:**

An observational study was conducted in a chronic wound care clinic involving 100 patients. The mean age was 68.02 ± 10.0 years. All participants had hard-to-heal wounds of vascular origin. The mean wound duration was 7.16 ± 5.08 months, with an average wound area of 39.18 ± 71.83 cm^2^ (range 2 cm^2^ to 625 cm^2^). Patients were randomly assigned to a group distracted with VR goggles and a control group receiving standard care without VR. Pain intensity was assessed using the Numeric Rating Scale (NRS) at three time points, and the McGill Pain Questionnaire (MPQ) before the procedure, during wound debridement, and 10 min after completion.

**Results:**

Statistically significant differences were observed in pain assessment before and during wound debridement (*p* < 0.05). In the VR group, higher pain scores were recorded before wound care compared to the control group. Ten minutes before wound debridement, the mean pain intensity in the VR group was 2.60 ± 1.63, higher than 2.0 ± 1.53 in the control group. During wound debridement, pain intensity was higher in the control group (4.94 ± 1.53) compared to the VR group (4.32 ± 2.17). Pain intensity 10 min after debridement was similar in both groups: control (2.24 ± 1.41) and VR (2.36 ± 1.71). These findings support the hypothesis that VR goggles reduce pain intensity. No statistically significant differences in NRS pain scores were found between patients with different wound types in either group (*p* > 0.05). Variables such as wound duration and wound size influenced pain levels 10 min before wound care. No association was found between sex and pain intensity (*p* > 0.005).

**Conclusion:**

Increased pain during procedures involving manipulation of damaged tissues and wound debridement is a common phenomenon. This study confirmed that the use of VR goggles reduces perceived pain levels. The assessment of pain experience and intensity varies depending on the assessment tools used; therefore, a combined quantitative and qualitative evaluation is recommended to accurately determine the usefulness of innovative tools in clinical practice.

## 1 Introduction

In recent years, there has been a steadily growing interest in modern technologies in medicine, which are opening new avenues for non-pharmacological support in alleviating acute pain associated with medical procedures, as well as for multimodal chronic pain management. One of the most promising technologies in this regard is Virtual Reality (VR), which is being increasingly applied across various areas of medicine, including the treatment of chronic wounds and the mitigation of procedural pain (1). In the medical context, Virtual Reality is defined as a three-dimensional, computer-generated environment, often delivered through a Head-Mounted Display (HMD), which fully immerses the user in a digital world and enables interaction with the generated surroundings (2).

Debridement, i.e., the removal of necrotic tissue, biofilm, and inflammatory debris from wounds, is a key component of the management of non-healing wounds, including those of vascular origin such as venous, arterial, and mixed pressure injuries. While this procedure offers significant therapeutic benefits—accelerating granulation tissue formation and reducing infection risk—it is frequently associated with severe, difficult-to-control pain (3, 4). Pain resulting from mechanical stimulation of the wound bed is often a major factor limiting patient cooperation and adversely affecting quality of life. Physical and psychological preparation, including education and basic psychological support, is particularly important in out-of-hospital settings where infiltration anesthesia options are limited (5, 6). Among patients receiving non-institutional care (long-term, hospice, or outpatient care), non-pharmacological pain-relief methods are of particular importance due to restricted access to specialized analgesics. An effective chronic wound management approach should focus not only on clinical aspects (e.g., selecting the appropriate debridement method) but also on patient comfort, engagement, and psychological well-being—by reducing distress related to dressing removal and wound preparation for re-dressing. These interventions align with the wound hygiene concept, whereby refreshing the wound bed and eliminating devitalized tissue reduces inflammation and strongly predicts healing outcomes (7, 8). In conventional clinical practice, procedural pain management during debridement relies primarily on pharmacological agents such as opioids and nonsteroidal anti-inflammatory drugs. However, these medications may cause adverse effects, especially in elderly patients who constitute the majority of those with vascular wounds. This has prompted the search for non-pharmacological solutions that are safe, effective, and suitable for home-based use. In this context, the implementation of innovative, non-invasive technologies—such as Virtual Reality—may serve as a valuable adjunct to standard interventions (9).

As a psychological intervention tool, Virtual Reality shows potential in modulating pain perception through mechanisms such as distraction and sensory immersion. Research indicates that patients undergoing VR therapy during painful procedures report lower pain intensity and improved psychological comfort (9, 10). This technology allows for the creation of interactive, engaging environments that divert the patient’s attention from painful stimuli to visually and acoustically attractive content. A growing body of evidence suggests that VR can be an effective tool for pain reduction in clinical care. In studies involving individuals with chronic pain, the use of VR during care procedures significantly reduced pain intensity and anxiety without the need for increased analgesic dosages. Such findings open new possibilities for holistic patient care (11, 12).

In non-institutional care settings, the acceptability and usability of VR technology by both patients and healthcare professionals are of critical importance. VR interfaces are becoming increasingly intuitive, and the devices themselves more affordable and portable, enabling easy integration into care environments (13). Moreover, some VR systems have already been adapted for use with telemedicine platforms, allowing for real-time monitoring of therapeutic outcomes and content adjustment. A crucial dimension of VR research in the context of vascular wounds is the psychological aspect. Chronic pain, fear of procedures, and limited mobility contribute to social isolation and depression among many patients with venous ulcers. Virtual Reality can serve not only as an analgesic tool but also as a motivational and emotionally supportive intervention (14). Interactive VR environments have been shown to improve mood, reduce anxiety, and enhance the overall perception of the treatment process. Given the new opportunities afforded by VR, a research initiative was undertaken to assess pain intensity using two standardized scales (the McGill Pain Questionnaire—MPQ and the Numeric Rating Scale—NRS) during the application of VR as a non-pharmacological intervention in the debridement of chronic, non-healing vascular wounds.

## 2 Materials and methods

### 2.1 Ethics

The study received a favorable opinion from the Bioethics Committee of the University of Rzeszów, as documented in Resolution No. 054/12/2024 dated December 20, 2024. Additionally, approval for the study was granted by the Director of the Specialist Hospital, Podkarpackie Oncology Center in Brzozów. The entire research process was conducted in accordance with the principles of the Declaration of Helsinki, ensuring informed participation and the right to withdraw from the study at any stage without pressure from the researcher, without the need to provide a reason for withdrawal, and with the guarantee that participants would continue to receive standard medical care appropriate to their condition (15).

### 2.2 Study objective

The aim of the study was to assess pain using two scales (MPQ and NRS) during the debridement of hard-to-heal wounds of vascular origin using surgical tools (sharp debridement performed by nursing staff), by applying VR as an adjunctive method of non-pharmacological pain relief. A prospective quantitative-qualitative study design was used, based on the observation of 100 patients selected according to established inclusion and exclusion criteria. The study was conducted in the largest facility in southeastern Poland providing services in the treatment of chronic and non-healing wounds, selected due to the general availability of free healthcare services for insured individuals and its ability to care for a sufficient number of patients to determine a sample size necessary to detect differences. To this end, a power analysis was conducted and calculations were performed using G*Power software with the following assumptions: effect size d = 0.6; alpha = 0.05; power = 0.8. The analysis showed that a minimum sample size of 47 participants was necessary to obtain reliable results. The study was conducted over a four-month period from December 2024 to March 2025 at the Wound Treatment Outpatient Clinic of the Podkarpackie Oncology Center in Brzozów. Participation in the study was offered to patients receiving treatment for chronic and hard-to-heal wounds of vascular origin at this facility.

**PHOTO 1:**
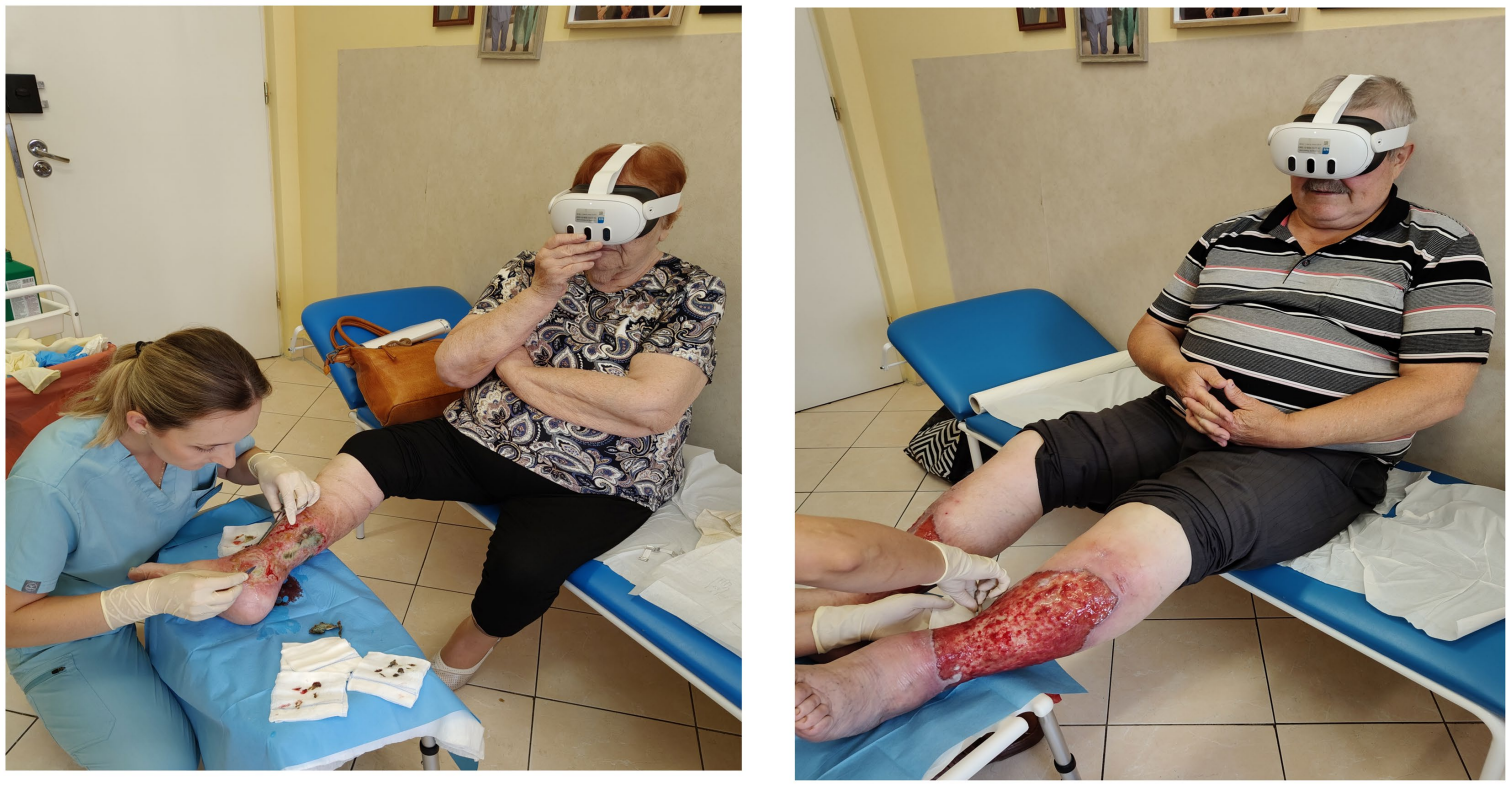
Use of VR during sharp debridement of vascular etiology wounds in the context of outpatient nursing care.

A purposive sampling approach was used, which meant that all clinic visits over the 6 months preceding the study (2,521 visits) involving 365 patients were analyzed. Patients with cancer-related wounds, ongoing wound and skin infections, pressure injuries, and improperly healing postoperative wounds were excluded from further consideration. This selection process identified a group of 123 patients who met the established inclusion criteria for the study sample. These criteria included the presence of venous ulceration classified as active, full preservation of auto- and allopsychic orientation, ability to communicate and express emotions, with wound area >10 cm^2^, and patients with diabetic foot disease (DFD) meeting the following conditions: wound area >2 cm^2^, WIFI >1, wound duration of at least 4 weeks and no more than 18 months, and pain level assessed on the NRS scale <4. In the case of patients with diabetic foot disease, individuals with smaller wound areas were included; however, due to the aggressive nature of the disease, these patients were at high risk of deep tissue and bone damage. Patients with wounds of non-vascular etiology and those reporting pain experiences >4 on the NRS scale were excluded from the study, including individuals presenting extreme pain sensations accompanied by excessive emotional responses (*n* = 6). Additional exclusion criteria included active inflammatory conditions or immunological disorders confirmed by the following findings: body temperature >38 °C, heart rate >90 beats/min, leukocytosis >12,000 or <4,000. The application of these inclusion and exclusion criteria resulted in the selection of 123 patients; however, 23 patients were ultimately disqualified due to analgesia, and none of the enrolled patients declined participation in the study, meaning they did not report any pain experience—even minimal—during sharp wound debridement (tissue manipulation with a surgical curette and scissors, debridement using a wound pad, or during surgical necrectomy). Ultimately, questionnaires from 100 patients who provided informed consent to participate after receiving complete information about the study objectives and procedures were included in the statistical analysis. None of the enrolled participants had uncorrected visual or hearing impairments, nor age-related conditions that would have prevented them from providing informed consent to participate in the study. The main study involved observing and comparing pain experiences occurring during the peri-procedural period (before starting the wound debridement, during the procedure, and 10 min after the medical intervention). Wound debridement and dressing changes were performed aseptically and gently, in accordance with current procedures, using topical anesthesia with 2% lidocaine gel applied to the surface of the skin and damaged tissues 3–5 min before starting the intervention. A simple randomization method was employed. Patients were randomly assigned (using the “random” function in Excel 2019) to Group A, which did not use VR goggles, and Group B, which used VR goggles during the wound debridement procedure. Each participant was informed of their group allocation. In Group B, VR goggles (Oculus Meta Quest 3, 128 GB, produced by Meta Platforms, Inc., Menlo Park, California, USA) were used for attention distraction during the wound care procedures. During the study, short films selected according to patient preferences were shown with accompanying sound. The films focused on nature and architectural themes and included one of four options: exotic beaches, forest trails, the underwater world, and the most fascinating buildings and landmarks in the world. VR projections began a few minutes before the start of the wound debridement and dressing change procedure and lasted until the end of the intervention, which in some cases required the looping of the video to play multiple times. During the study, the VR goggles were disinfected after each use according to established hospital procedures and the principles for disinfecting reusable equipment. None of the participants undergoing the VR sessions reported any adverse effects such as dizziness, eye fatigue, or nausea. Sample photos from the study are presented in Photo 1.

### 2.3 Data collection

The research protocol consisted of two parts. The first part involved collecting sociodemographic data (gender, age, place of residence, marital status, education, and economic status), assessing self-care levels using the Barthel Index, and evaluating tissue injury characteristics (type, size, depth of the wound, and duration of pathological changes). Wound assessment employed a simple classification of partial-thickness skin injury, full-thickness injury, and damage involving deeper tissues, alongside the EPUP/NPIAP scale, WIfI, and RYB classifications. The second part gathered information on pain experiences in both groups A and B, using the Numeric Rating Scale (NRS) and the McGill Pain Questionnaire (MPQ, R. Melzack). Patients easily indicated pain intensity on the NRS (0 = no pain; 10 = worst possible pain), but given pain’s individual and subjective nature, qualitative assessment was also performed using the Polish-adapted McGill Pain Questionnaire by Kołłątaj et al. (16, 17). This instrument assesses sensory (groups 1–10), emotional (groups 11–15), and subjective (group 16) pain dimensions, along with additional categories (groups 17–20). Participants were instructed to read all descriptors in each group at least once and select only those accurately describing their pain. The questionnaire characterized pain by the number of words chosen (NWC), the pain rating index (PRI) based on mean and rank values, and the Present Pain Intensity (PPI) on a six-point scale (0 = no pain; 5 = excruciating pain). It also included items assessing pain’s impact on daily activities, sleep, and nutrition, and characterized pain temporal patterns (continuous, intermittent, momentary). Patients could indicate pain location on a front-and-back human figure diagram.

### 2.4 Statistical analysis

Statistical analysis was conducted using IBM SPSS Statistics version 21. To accurately assess relationships between variables, descriptive statistics, histograms, and boxplots were employed, alongside Kolmogorov–Smirnov tests for normality of distributions. Spearman’s rank-order correlation (rho), Kruskal–Wallis tests, and Mann–Whitney U tests were used to evaluate differences in the distributions of dependent variables across categories of independent variables.

## 3 Results

### 3.1 Characteristics of respondents

The mean age of participants was 68.02 ± 10.0 years, with a median of 70; in group A (without VR goggles), the mean age was 67.46 ± 11.27, median 67, and in group B (with VR goggles), 68.58 ± 8.63, median 70. The age range for the entire cohort was 43 to 89 years (group A: 43–89 years, group B: 46–85 years). Over 30% of all participants were aged up to 64 years, 45.0% were between 65 and 74 years old, and 25.0% were older than 75 years. The gender distribution was nearly equal, with 51% female and 49% male participants (group A—females 46.0%, males 54.0%; group B—females 56.0%, males 44.0%). Regarding place of residence, 62.0% of participants lived in rural areas, while 38.0% were urban residents (group A: city 48.0%, rural 52.0%; group B: city 28.0%, rural 72.0%). Respondents were assessed for self-care ability using the Barthel Index, achieving an overall mean score (M ± SD) of 89 ± 12.53, with a median of 90 (group A: 88.2 ± 13.69, median 90; group B: 89.80 ± 11.34, median 90). Across the entire cohort and within groups A and B, Barthel Index scores ranged from 40 to 100. The majority of participants (61.0%) were classified as self-care competent, scoring between 86 and 100 (group A—62.0%, group B—60%), while 39.0% exhibited mild self-care deficits, scoring between 21 and 85 (group A—38.0%, group B—40.0%). None of the participants were classified as unable to perform self-care. The use of the McGill Pain Questionnaire also allowed for the assessment of subjective symptoms accompanying pain experienced by participants, as well as the impact of pain on daily activity, sleep, and nutrition. In the entire group, 89.0% reported good nutrition, followed by 6.0% reporting reduced nutrition and 2.0% reporting limited nutrition. Seventy percent reported good sleep quality, 25.0% experienced restless sleep, and 3.0% complained of insomnia.

### 3.2 Characteristics of wounds and pain treatment

In the studied cohort, venous ulcers predominated (49.0%), followed by mixed ulcers (35.0%), diabetic foot ulcers (DFU) (10%), and arterial ulcers (6.0%). The mean duration of ulcer existence was 7.16 ± 5.08 months, with a median of 5 months (group A: 5.92 ± 4.49 months, median 4; group B: 8.40 ± 5.36 months, median 7). Analysis of ulcer location revealed the following distribution: medial part of the lower leg (55.0%), lateral part of the lower leg (25.0%), foot (14.0%), and circumferential lesion on the lower leg (6.0%). The average wound size in cm^2^ was 39.18 ± 71.83, with a median of 17 (group A: 23.61 ± 30.29, median 15; group B: 54.74 ± 94.94, median 29). The wound area ranged from 2 to 625 cm^2^ overall (group A: 3–150 cm^2^; group B: 2–625 cm^2^). Tissue destruction analysis according to the NPIAP classification showed a mean score of 2.68 ± 0.62, median 3, indicating that over half of the patients (52.0%) had full-thickness skin ulcers (grade 3 tissue destruction). Partial-thickness skin wounds (grade 2 NPIAP) accounted for 40.0% of the group, and 8.0% had wounds penetrating deeper tissues (grade 4). The wound assessments according to the NPIAP, RYB, and WIfI classifications are presented in Table 1.

**Table 1:** Wound assessment according to NPIAP, RYB, and WIfI classifications.

	Total Group		Group A	Without VR Goggles	Group B	With VR Goggles
	*n*	%	*n*	%	*n*	%
Tissue destruction assessment according to NPIAP classification						
Grade 2 (partial-thickness skin damage)	40	40.0%	22	44.0%	18	36.0%
Grade 3 (full-thickness skin damage)	52	52.0%	24	48.0%	28	56.0%
Grade 4 (penetrating wound)	8	8.0%	4	8.0%	4	8.0%
Total	100	100.0%	50	100.0%	50	100.0%
Wound assessment using the RYB scale						
Red	8	8.0%	7	14.0%	1	2.0%
Red-yellow	67	67.0%	31	62.0%	36	72.0%
Yellow	22	22.0%	11	22.0%	11	22.0%
Black	3	3.0%	1	2.0%	2	4.0%
Total	100	100.0%	50	100.0%	50	100.0%
WIfI assessment						
1	1	1.0%	1	2.0%	0	0.0%
2	11	11.0%	5	10.0%	6	12.0%
3	0	0.0%	0	0.0%	0	0.0%
Not specified	88	88.0%	44	88.0%	44	88.0%
Total	100	100.0%	50	100.0%	50	100.0%

All patients included in the study underwent wound cleansing prior to dressing application. Among the selected debridement methods, scraping was the most commonly used (overall 70.0%, group A 68.0%, group B 72.0%), followed by scraping combined with removal of devitalized tissue (overall 27.0%, group A 30.0%, group B 24.0%), and necrosectomy (overall 3.0%, group A 1.0%, group B 2.0%). Nearly three-quarters of the vascular ulcer cohort used analgesics on an as-needed basis (overall 72.0%, group A 74.0%, group B 70.0%), while 28.0% of the total cohort took analgesics regularly and in a planned manner (group A 26.0%, group B 30.0%). The majority used first-step analgesics from the WHO analgesic ladder, most commonly over-the-counter drugs (75.0%). Weak opioids combined with other non-opioid drugs were used by 23.0% of respondents, whereas third-step analgesics were prescribed to 2.0% of patients. Analysis of patients’ pain experiences and their impact on other daily activities, as reported in the MPQ questionnaire before wound treatment, showed that most participants complained of intermittent pain (54.0%), momentary pain (40.0%), and continuous pain (5.0%).

### 3.3 Pain symptoms during wound cleansing

Pain intensity was assessed using the NRS and MPQ scales at two time points: 10 min before wound debridement and during the wound cleansing procedure. The mean pain intensity in the entire cohort 10 min before the procedure was 2.30 ± 1.60, with a median of 2 (group A: 2.0 ± 1.53, median 2; group B: 2.60 ± 1.63, median 3). During wound cleansing, pain intensity increased overall to a mean of 4.63 ± 1.89 (group A: 4.94 ± 1.53, median 5; group B: 4.32 ± 2.17, median 4). Significant differences in pain assessment between groups A and B were observed both 10 min before and during wound cleansing. Ten minutes prior, the median pain score was higher in group B than in group A; however, during the procedure, this trend reversed, with group A reporting higher median pain scores than group B. These findings suggest that focusing attention on a VR-displayed video and sparing patients from unpleasant visual stimuli such as devitalized tissue, pus, and blood reduces pain perception and helps alleviate pain during wound cleansing and dressing changes. The results are presented in Table 2.

**Table 2:** Pain intensity measured by NRS 10 minutes before and during wound cleansing in the overall cohort and groups A and B.

	Assessment 10 min before wound cleansing			Assessment during wound cleansing		
	Total	Group A—without VR goggles	Group B—with VR goggles	Total	Group A—without VR goggles	Group B—with VR goggles
Mean	2.30	2.00	2.60	4.63	4.94	4.32
SD	1.60	1.53	1.63	1.89	1.53	2.17
Median	2	2	3	5	5	4
Min	0	0	0	0	2	0
Max	4	4	4	10	8	10
Q1	1	1	1	4	4	3
Q3	3	3	4	5	6	5
n	100	50	50	100	50	50

**Figure 1:**
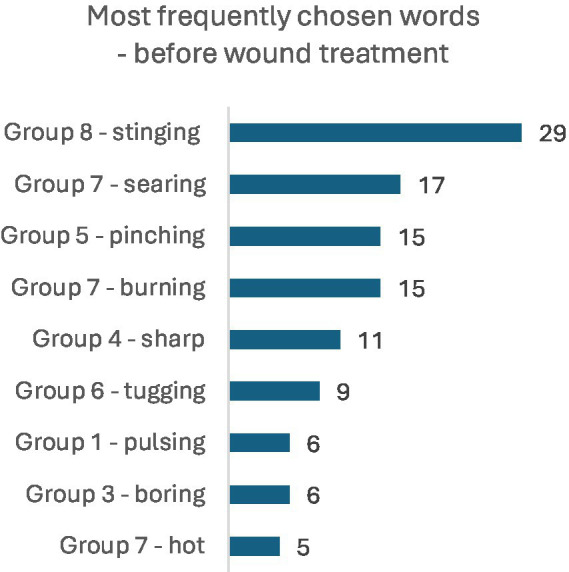
Most frequently chosen words in the MPQ 10 min before wound treatment (NWC).

A similar assessment was conducted using the McGill Pain Questionnaire developed by R. Melzack at identical time points—10 min before wound treatment and during the cleansing process. The questionnaire consists of multiple components, each evaluated separately. The Pain Rating Index (PRI) before the medical intervention averaged 3.68 ± 3.35 with a median of 3 in the entire group (Group A: 3.36 ± 2.75; Group B: 4.0 ± 3.87). During wound treatment, the PRI averaged 3.49 ± 2.66 with a median of 3 overall (Group A: 3.9 ± 3.08; Group B: 3.08 ± 2.13). Results are presented in Table 3 and illustrated by a boxplot (Figure 1).

**Table 3:** Pain rating index (PRI) 10 minutes before and during wound treatment.

	Assessment 10 min before wound treatment			Assessment during wound treatment		
	Total	Group A—without VR goggles	Group B—with VR goggles	Total	Group A—without VR goggles	Group B—with VR goggles
Mean	3.68	3.36	4.00	3.49	3.90	3.08
SD	3.35	2.75	3.87	2.66	3.08	2.13
Median	3	3	3	3	3	3
Min	0	0	0	0	0	0
Max	24	9	24	12	12	8
Q1	2	1	2	2	2	2
Q3	6	5	6	5	6	4
n	100	50	50	100	50	50

The next evaluated indicator was the Number of Words Chosen (NWC) to describe pain experiences. Ten minutes before wound cleansing, the overall mean in this category was 1.59 ± 1.16, with a median of 2 (Group A: 1.54 ± 1.09, median 1; Group B: 1.64 ± 1.22, median 2). During wound cleansing, the overall mean NWC value was 1.55 ± 0.93, with a median of 1 (Group A: 1.80 ± 1.01, median 2; Group B: 1.30 ± 0.78, median 1). The results are presented in Table 4.

**Table 4:** Number of words chosen (NWC) at 10 minutes before and during wound cleansing.

	Assessment 10 min before wound treatment			Assessment during wound treatment		
	Total	Group A—without VR goggles	Group B—with VR goggles	Total	Group A—without VR goggles	Group B—with VR goggles
Mean	1.59	1.54	1.64	1.55	1.80	1.30
SD	1.16	1.09	1.22	0.93	1.01	0.76
Median	2	1	2	1	2	1
Min	0	0	0	0	0	0
Max	8	4	8	4	4	3
Q1	1	1	1	1	1	1
Q3	2	2	2	2	2	2
n	100	50	50	100	50	50

Based on the Kolmogorov–Smirnov normality test, it can be concluded that the distributions of the PRI and NWC variables significantly differ from a normal distribution (Table 5).

**Table 5:** Kolmogorov–Smirnov normality tests of PRI and NWC distributions 10 minutes before and during wound treatment in groups A and B.

	Group	Kolmogorov–Smirnov		
		Statistic	df	Significance
Pain Rating Index—10 min before	Group A—no VR goggles	0.132	50	0,029
	Group B –VR goggles	0.179	50	0.000
Pain Rating Index—during	Group A—no VR goggles	0.195	50	0.000
	Group B—VR goggles	0.154	50	0.005
Number of Words Chosen—10 min before	Group A—no VR goggles	0.210	50	0.000
	Group B—VR goggles	0.304	50	0.000
Number of Words Chosen—during	Group A—no VR goggles	0.246	50	0.000
	Group B—VR goggles	0.293	50	0.000

In the statistical evaluation using the Mann–Whitney U test, the comparison of PRI distributions 10 min before and during wound treatment between groups A and B showed no statistically significant differences at either time point. However, for the NWC, no significant difference was found between groups A and B in the distribution of selected words 10 min before wound treatment (*p* > 0.05). A significant difference was observed in the distribution of NWC during wound treatment (*p* = 0.016). The median in group B (with VR goggles) was lower than in group A (without VR goggles), indicating that group B, on average, chose fewer words describing pain than group A. The data are presented in Table 6.

**Table 6:** Mann–Whitney U test statistics—comparison of PRI and NWC distributions 10 minutes before and during wound treatment between groups A and B.

	Pain rating index - 10 min before	Pain rating index - during	Number of words chosen - 10 min before	Number of words chosen - during
Mann–Whitney U	1150.000	1125.000	1201.000	923.500
Wilcoxon W	2425.000	2400.000	2476.000	2198.500
Z	−0.695	−0.871	−0.355	−2.420
Asymptotic significance (2-tailed)	0.487	0.384	0.722	0.016

Present Pain Intensity (PPI) assessed using the MPQ questionnaire 10 min before the examination indicates low pain intensity in the overall group (1.29 ± 0.87), in Group A (1.14 ± 0.76), and in Group B (1.44 ± 0.95). However, during the examination, an increase in pain intensity was recorded, with the mean value rising to 2.69 ± 0.97 in the overall group, 2.90 ± 0.91 in Group A, and 2.48 ± 0.97 in Group B. Very severe pain sensations were not confirmed in the MPQ assessment (Tables 7, 8).

**Table 7:** MPQ present pain intensity (PPI) before wound treatment in groups A and B.

CPI	Group					
	Total		A - no VR Goggles		B - VR Goggles	
	n	%	n	%	n	%
0-no pain	16	16.0%	9	18.0%	7	14.0%
1-mild	50	50.0%	27	54.0%	23	46.0%
2-discomforting	23	23.0%	12	24.0%	11	22.0%
3-distressing	11	11.0%	2	4.0%	9	18.0%
4-horrible	0	0.0%	0	0.0%	0	0.0%
5-exruciating	0	0.0%	0	0.0%	0	0.0%
Total	100	100.0%	50	100.0%	50	100.0%

**Table 8:** MPQ present pain intensity (PPI) during wound treatment in groups A and B.

PPI	Group					
	Total		A - no VR Goggles		B - VR Goggles	
	n	%	n	%	n	%
0-no pain	3	3.0%	1	2.0%	2	4.0%
1-mild	9	9.0%	4	8.0%	5	10.0%
2-discomforting	22	22.0%	5	10.0%	17	34.0%
3-distressing	48	48.0%	29	58.0%	19	38.0%
4-horrible	18	18.0%	11	22.0%	7	14.0%
5-exruciating	0	0.0%	0	0.0%	0	0.0%
Total	100	100.0%	50	100.0%	50	100.0%

A comparison was made of pain experiences measured by the NRS scale and collected using the MPQ questionnaire. In both groups, fairly high and statistically significant correlation coefficients were observed between the NRS scores and the Pain Rating Index (PRI) as well as the Number of Words Chosen (NWC) before the procedure. The obtained data indicate that assessments made prior to the procedure are consistent (*p* < 0.05). However, pain measurements taken during wound treatment show divergence when comparing these tools. The correlation coefficients of the analyzed variables during the procedure are low, yet statistically significant (*p* < 0.05), especially in the area of Present Pain Intensity (PPI). The data are presented in Table 9.

**Table 9:** Spearman’s rho correlation coefficients between NRS and MPQ (PPI, NWC, and PRI) in groups A and B before and during the procedure.

*n* = 50 per group	NRS 10 min before the procedure		NRS during the procedure	
	Spearman’s rho	Sig (2-sided)	Spearman’s rho	Sig (2-sided)
Group A				
PPI (Present Pain Intensity) 10 min before the procedure	0.587	0.000		
PPI (Present Pain Intensity) during the procedure			0.358	0.011
PRI (Pain Rating Index) 10 min before the procedure	0.660	0.000		
PRI (Pain Rating Index) during the procedure			0.215	0.134
NWC (Number of Words Chosen) 10 min before the procedure	0.641	0.000		
NWC (Number of Words Chosen) during the procedure			0.259	0.069
Group B				
PPI (Present Pain Intensity) 10 min before the procedure	0.906	0.000		
PPI (Present Pain Intensity) during the procedure			0.774	0.000
PRI (Pain Rating Index) 10 min before the procedure	0.639	0.000		
PRI (Pain Rating Index) during the procedure			0.253	0.076
NWC (Number of Words Chosen) 10 min before the procedure	0.578	0.000		
NWC (Number of Words Chosen) during the procedure			0.185	0.199

**Figure 2:**
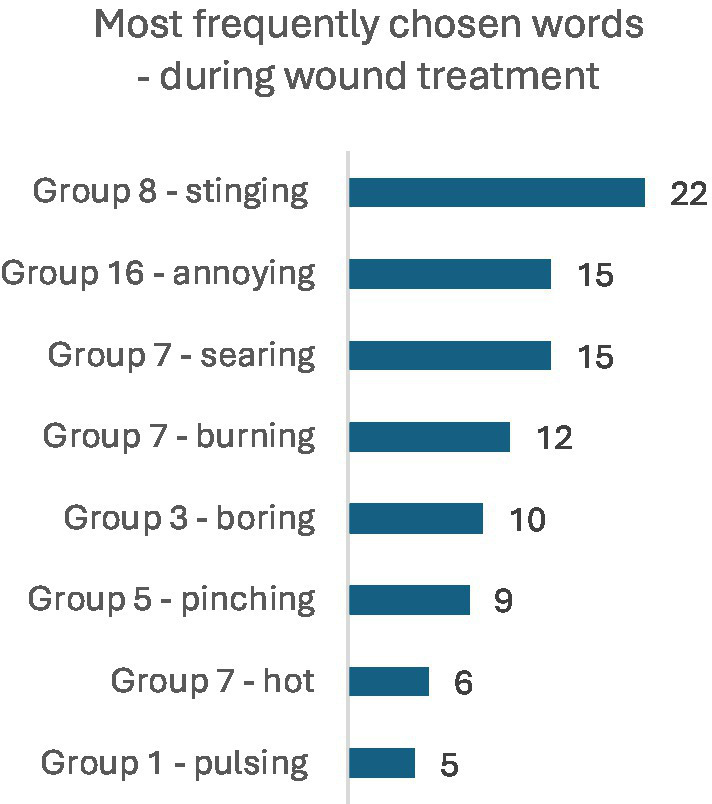
Most frequently chosen words in the MPQ during wound treatment (NWC).

Among the most frequently chosen words describing pain in the MPQ 10 min before wound treatment, the dominant terms were: stinging (*n* = 29), searing (*n* = 17), pinching and burning (*n* = 15), and sharp (*n* = 11). Next in frequency were: tugging (*n* = 9), pulsing and boring (6 persons each), and hot (*n* = 5). The set of selected words changed during wound treatment, with the most commonly chosen words being: stinging (*n* = 22), annoying and searing (15 persons each), burning (*n* = 12), boring (*n* = 10), pinching (*n* = 9), hot (*n* = 6), and pulsing (*n* = 5). Detailed results are illustrated in the figures. The set of pain descriptors indicates the presence of pain of a neuralgia type (Figures 1, 2).

## 4 Discussion

In recent decades, intensified research on pain has led to the development of the biopsychosocial paradigm, followed by an updated definition of pain in 2020 by experts from the International Association for the Study of Pain (IASP). Pain is now defined as “an unpleasant sensory and emotional experience associated with actual or potential tissue damage” (18). The recognition of pain as a subjective experience influenced by both biological and psychosocial factors introduces certain challenges in assessment—particularly in cases involving individual perception and confounding factors such as older age, gender, and personal constitutional characteristics. In the present study, which examined the potential use of virtual reality (VR) during wound cleansing procedures, several interesting variables and distracting factors were observed that may influence pain intensity ratings (10). It was noted that individuals with ischemic wounds, women, and those reporting fatigue and restless sleep demonstrated a heightened pain threshold. Subjective assessments of pain were conducted using two clinical scales (NRS and MPQ). Additional statistical analyses were carried out to explore the main research aim: investigating the relationship between patient distraction using an advanced VR system and the experience of potential pain before and during the cleansing of vascular wounds.

Over the past decade, researchers have increasingly pointed to virtual reality (VR) in the context of emotional, affective, and cognitive responses that influence the complex system of pain modulation (19–21). In an effort to understand the mechanistic origin of VR-induced analgesia, analyses have focused on the neurobiological interaction between cortical brain activity and neurochemistry, as well as processes related to attention, emotional regulation, and cognition. One of the significant milestones in this area is the Gate Control Theory proposed by Melzack and Wall, which suggested a correlation between levels of attention and emotion related to pain and the subsequent perception and interpretation of pain experiences (22). An extension of this theory came from McCaul and Malott, who argued that due to the limited capacity of attention, a person must focus on a stimulus for it to be perceived as painful. This led to the hypothesis that if one’s attention is engaged elsewhere, painful stimuli—although present—will be perceived as less intense (23). A further breakthrough was the Multiple Resources Theory introduced by Wickens, which demonstrated the independent functioning of resources across different sensory systems. These findings support the rationale for using multimodal distractions (visual, auditory, tactile, and olfactory) through VR technologies and their implementation in medical practice (24).

In a prospective observational study conducted within a wound care outpatient clinic, Oculus Meta Quest® 3 goggles were used during the procedure of wound debridement and dressing (scraping and removal of necrotic tissue). Of the 123 patients with chronic vascular wounds, 100 were included in the analysis: 49.0% (*n* = 49) men and 51.0% (*n* = 51) women. The participants’ ages ranged from 43 to 89 years, with a mean age of 68.02 ± 10.0 years. During the qualification process, patients were randomly assigned to two groups: Group B (with VR goggles) and Group A (without goggles). Prior to the procedure, all patients received lignocaine gel for local anesthesia of the wound bed. The majority had venous (49.0%) or mixed etiology wounds (35.0%), most commonly located on the medial (55.0%) or lateral (25.0%) aspects of the lower leg. Most participants were self-sufficient in daily care (61.0%), while 39.0% presented with minor deficits. Only 28.0% of patients were on regular analgesic treatment; the remainder used painkillers occasionally in response to increasing pain. Before the therapeutic procedures, pain intensity did not exceed 4 points on the NRS, with a mean of 2.3 ± 1.8 across both groups. Pain intensity was assessed using two scales: the MPQ and the NRS. The MPQ questionnaire was used before and during the wound debridement, while the NRS scale was applied for direct subjective assessment before, during, and 10 min after the procedure. According to NRS scores, participants in the VR group (B) reported lower pain intensity before the procedure (2.0 ± 1.53) compared to the non-VR group (A) (2.6 ± 1.63). During the procedure, lower pain ratings were again recorded in Group B (4.32 ± 2.17) compared to Group A (4.94 ± 1.53) (*p* < 0.05). Ten minutes after the procedure, a reduction in pain intensity was observed: 2.24 ± 1.41 in Group A and 2.36 ± 1.71 in Group B. In the VR group, the median NRS pain score increased by 3 points during the procedure, whereas in the non-VR group, it increased by only 1 point. The McGill Pain Questionnaire is a quantitative and qualitative tool used to assess pain of various etiologies. It consists of 20 pain descriptors (Number of Words Chosen—NWC), a Pain Rating Index (PRI), and a measure of Present Pain Intensity (PPI).

Among the most frequently chosen words describing pain in the McGill Pain Questionnaire 10 min before wound care, the predominant descriptors were: stinging (*n* = 29), searing (*n* = 17), pinching and burning (*n* = 15), and sharp (*n* = 11). The next most frequent descriptors included: tugging (*n* = 9), pulsing and boring (*n* = 6), and hot (*n* = 5). The repertoire of chosen words changed during the wound care procedure, with the most frequently selected descriptors being: stinging (*n* = 22), annoying (*n* = 15), searing (*n* = 15), burning (*n* = 12), boring (*n* = 10), pinching (*n* = 9), hot (*n* = 6), and pulsing (*n* = 5). The range of words describing pain in patients’ assessments indicates the presence of pain associated with nerve ending irritation, suggestive of neuralgia. The pain assessment index before the start of the medical intervention in the entire study group averaged 3.68 ± 3.35, median 3 (in group A: 3.36 ± 2.75, in group B: 4.0 ± 3.87), whereas during wound care the Pain Rating Index (PRI) was 3.49 ± 2.66, median 3 (group A: 3.9 ± 3.08, group B: 3.08 ± 2.13). Statistical evaluation using the Mann–Whitney U test showed no significant differences in Pain Rating Index (PRI) distributions between groups A and B at the two time points (*p* > 0.05). Similarly, no statistically significant differences were observed between groups A and B in the distribution of the Number of Words Chosen (NWC) 10 min before wound care (*p* > 0.05). However, significant differences were found in the distribution of NWC during wound care (*p* = 0.016). The median NWC in group B (with VR goggles) was lower than in group A (without VR goggles), indicating that group B on average selected fewer pain descriptors than group A. Considering parameters defining Present Pain Intensity (PPI), very interesting observations were made based on the Likert scale assessment (no pain, mild, discomforting, distressing, horrible, excruciating). A comparison was made between pain sensation data measured by the Numeric Rating Scale (NRS) and data collected via the MPQ questionnaire. Both groups showed relatively high and statistically significant correlation coefficients between the NRS score and the Pain Rating Index (PRI), as well as the Number of Words Chosen (NWC) before the procedure. These data indicate consistency in pre-procedure assessments (*p* < 0.05). However, pain measurements conducted during wound care showed discrepancies between these tools. The correlation coefficients for the analyzed features during the procedure remained statistically significant (*p* < 0.05), especially in the PPI domain. Although the assessments using the two scales differ, they clearly indicate lower pain experiences in the group where VR goggles were used. Assessment of pain intensity using numerical and analog scales is straightforward but not without errors, as it represents subjective perception of negative sensations, which is individual to each patient. Researchers studying pain intensity phenomena should be aware that patients may overestimate or underestimate pain by reporting extreme values depending on the context. In our study, several extreme cases related to pain sensations were excluded, specifically 3 women reporting pain intensity above 10 points without typical symptoms indicating very severe pain stimuli. The correlation between NRS pain scores and MPQ questionnaire assessment was low. Similar observations were reported by Nugent et al. in a study evaluating somatic pain in a group of opioid users; the authors concluded that standardized pain assessments during care are moderately correlated with research-administered pain intensity measures and could be improved by incorporating more robust measures of pain-related functioning, mental health, and quality of life in health (25). Despite the interesting confirmed observations related to the assessment of pain intensity, the present study has certain limitations. In our research, no adjustment was made for confounding variables affecting pain experiences, such as emotional state. Although, in the overall study, anxiety as a trait and as a state was assessed, these data were not presented in this material (no association was found between pain and the severity of anxiety as a trait or as a state). Cognitive functions of the participants were also not assessed in detail, although the inclusion criteria specified the level of communication and ability to cooperate. Multivariable models were likewise not used to control for other variables influencing pain. During the course of the study, it was observed that individuals living alone, without support or assistance, exhibited stronger emotional reactions compared to the rest of the group. These variables may affect the reliability of the results. Although medical history regarding analgesic use was obtained and pain sensations were assessed, such parameters may be insufficient.

One of the most extensively studied applications of VR technology in alleviating pain and anxiety during medical interventions and rehabilitation processes has been research involving burn patients. In a randomized controlled trial, Das et al. demonstrated the superior effectiveness of analgesia combined with VR in reducing pain and distress during procedures in burn-injured children aged 5 to 18 years compared to standard analgesia alone (26). The effectiveness of VR technology in pediatric burn care has also been confirmed in other studies by Hoffman et al. He investigated the use of VR during burn wound cleaning procedures conducted in a water environment and during physiotherapy sessions in burn patients, confirming that VR effectively reduces pain by engaging patients in play, as well as resulting in lower pain ratings and increased range of motion during physiotherapy when VR was used in addition to pharmacological anesthesia (27–29). Sharar et al. reported findings from three studies confirming reductions in pain intensity, unpleasantness, and the amount of time spent thinking about pain symptoms in patients who received VR combined with standard anesthesia (30). Similar results in groups of burn patients undergoing physiotherapy/rehabilitation were obtained by Carrougher et al. (31). In our study, VR was implemented with interesting effects in a group of patients with chronic wounds. This is one of the few recent studies conducted in Poland demonstrating the potential of VR use in outpatient care. Existing evidence of VR’s ability to reduce pain and anxiety during medical and rehabilitation procedures has broadened research interests toward the use of VR hypnosis (Virtual Reality Hypnosis, VRH), which has been shown to lower pain and anxiety levels (32–34). Our own results, obtained using the NRS and MPQ scales, justify the conclusion that the use of VR during wound care procedures reduces perceived pain intensity by distracting patients. However, assessments obtained via the individual tools are not consistent. This observation warrants further investigation in future studies, with particular attention to evaluations using the simple numerical scale (which tends to overestimate pain) and the more comprehensive quantitative-qualitative tool, the MPQ. Our study highlighted that qualitative assessment of pain intensity in scientific contexts is more desirable, as it precisely identifies symptoms and sensations rather than merely measuring pain intensity alone. In summarizing the conducted project, attention was also drawn to the costs associated with the procurement and maintenance of VR equipment in medical facilities providing inpatient, outpatient, and home care services. The purchase and upkeep of the equipment are commensurate with the potential benefits arising from increased patient satisfaction, which results from pain reduction through distraction and the alleviation of negative emotions related to observing the removal of dressings, wound cleansing procedures, including the sight of devitalized, necrotic tissue, pus, and blood. However, for VR-based procedures to become widespread and standard practice in Poland, public funding would be necessary. The broad implementation of this innovation would require further studies focusing on cost-effectiveness and feasibility analyses. It should be noted that VR can also be utilized for other ambulatory diagnostic procedures, especially in pediatric populations and individuals with heightened sensitivity, such as blood sampling for biochemical tests or other interventions where exposure to bodily excretions and secretions may induce anxiety and exacerbate negative emotional experiences.

Previous research on the use of VR in the treatment of vascular wounds has been limited in scale and often focused on hospital settings. Therefore, there is an urgent need to deepen knowledge regarding the potential application of VR during debridement procedures conducted outside the hospital, where organizational challenges, personnel shortages, and technical limitations affect the quality of care. This study addresses that knowledge gap by analyzing the effectiveness of VR in alleviating pain during the cleansing of vascular wounds in outpatient care settings.

## 5 Conclusion

The increase in pain experiences during procedures involving manipulation of damaged tissues and wound debridement is a well-known phenomenon. This study confirmed a reduction in perceived pain levels during the use of VR goggles, which occurred due to distraction of attention and limitation of the unpleasant view of devitalized tissues, pus, and blood. Pain sensation and intensity assessments differ depending on the selected evaluation tools; therefore, a combined quantitative and qualitative assessment of pain sensations and intensity is recommended to accurately indicate the usefulness of innovative tools in clinical practice. The use of VR as an adjunct strategy to reduce pain intensity and pain sensations during the management of hard-to-heal wounds may be recommended in healthcare; however, it requires further observational studies in various clinical situations.

## 6 Study limitations

A limitation of the study was the exclusion of patients whose pain level during their first visit to the wound care clinic, assessed using the NRS scale, exceeded 4 points. This criterion was adopted to prevent escalation of pain experiences during wound cleansing. Patients receiving strong opioid medications were also excluded due to the risk of data distortion, as well as individuals who recorded extreme pain intensity ratings during the procedure. A limitation of the study was the lack of control and monitoring of the medications regularly taken by patients, including analgesics, which might have influenced the level of perceived pain immediately before the intervention and after wound cleansing. Another limitation was the absence of physiological marker recordings, such as heart rate, blood pressure, and respiratory rate. Another limitation was data collection at only two time points—10 min before the wound care intervention and during the wound care procedure in groups A and B—without assessments conducted within 10 min after the intervention or at later times (e.g., 2 h post-intervention), which could have revealed associations with the persistence or normalization of patient pain symptoms. Future studies should consider analyzing the influence of mental, emotional, and social factors on pain experiences during wound cleansing interventions with and without VR. The study was conducted solely in outpatient settings (wound care clinic); no assessments were performed in home environments during chronic wound care. Considering the home environment in future research would provide valuable insights.

## Data Availability

The raw data supporting the conclusions of this article will be made available by the authors, without undue reservation.
